# Obstructive Coronary Atherosclerosis in a Patient with a Calcium Score of Zero

**DOI:** 10.14797/mdcvj.845

**Published:** 2021-09-24

**Authors:** Talal Alnabelsi, Faisal Nabi, Mouaz Al-Mallah

**Affiliations:** 1 Houston Methodist DeBakey Heart & Vascular Center, Houston, TX, USA

**Keywords:** PET, calcium score, obstructive disease

## Abstract

Museum images feature novel multimodality imaging that illuminates the important role of imaging in the diagnosis and management of on unusual cases and conditions. Image files should be accompanied by a contextual description of approximately 100 to 300 words. Videos are welcome, and abstracts are unnecessary. To submit museum images to be considered for peer review, please visit *journal.houstonmethodist.org*.

A 62-year-old female with morbid obesity, hypertension, and hyperlipidemia presented to the emergency department for evaluation of atypical substernal chest pressure and shortness of breath. The symptoms had lasted 6 to 8 weeks with a crescendo pattern. An electrocardiogram on admission showed normal sinus rhythm without ischemic changes. The 0- and 3-hour cardiac enzymes (troponin I) were minimally elevated without a significant delta. Given the patient’s low GRACE score, we pursued an ischemia-guided strategy and performed a positron emission tomography (PET) scan. The patient underwent a standard rest-stress pharmacologic (regadenoson) PET with rubidium-82. The stress echocardiogram showed no ischemic changes, and the ejection fraction (EF) did not increase at peak stress (rest EF 70%, stress EF 67%). The perfusion images revealed a large, moderate to severe intensity, completely reversible perfusion defect in the basal to distal anterior and antero-septal segments that was suspicious for ischemia in the left anterior descending (LAD) coronary artery vascular territory (***Figure 1A***). Surprisingly, the patient’s calcium score (performed as a standard part of the PET exam) was zero (***Figure 1B***). This raised concern of a false positive perfusion study and prompted us to reinspect the technical aspects of image acquisition; however, no technical errors were identified. Myocardial blood flow analysis revealed abnormal flow augmentation in the LAD territory (peak stress flow in the LAD 1.3 mL/min/g; expected normal > 1.8 mL/min/g) and normal flow augmentation in the rest of the coronary vessels (***Figure 1C***). The PET scan was abnormal with 26% ischemia in the LAD vascular territory. The patient underwent coronary angiography, revealing a 90% proximal LAD lesion, which was treated with a (3.0 × 15) Resolute Onyx drug-eluting stent (Medtronic; ***Figure 1D*** and ***1E***). The remaining coronary arteries were free of atherosclerotic disease.

**Figure 1:**
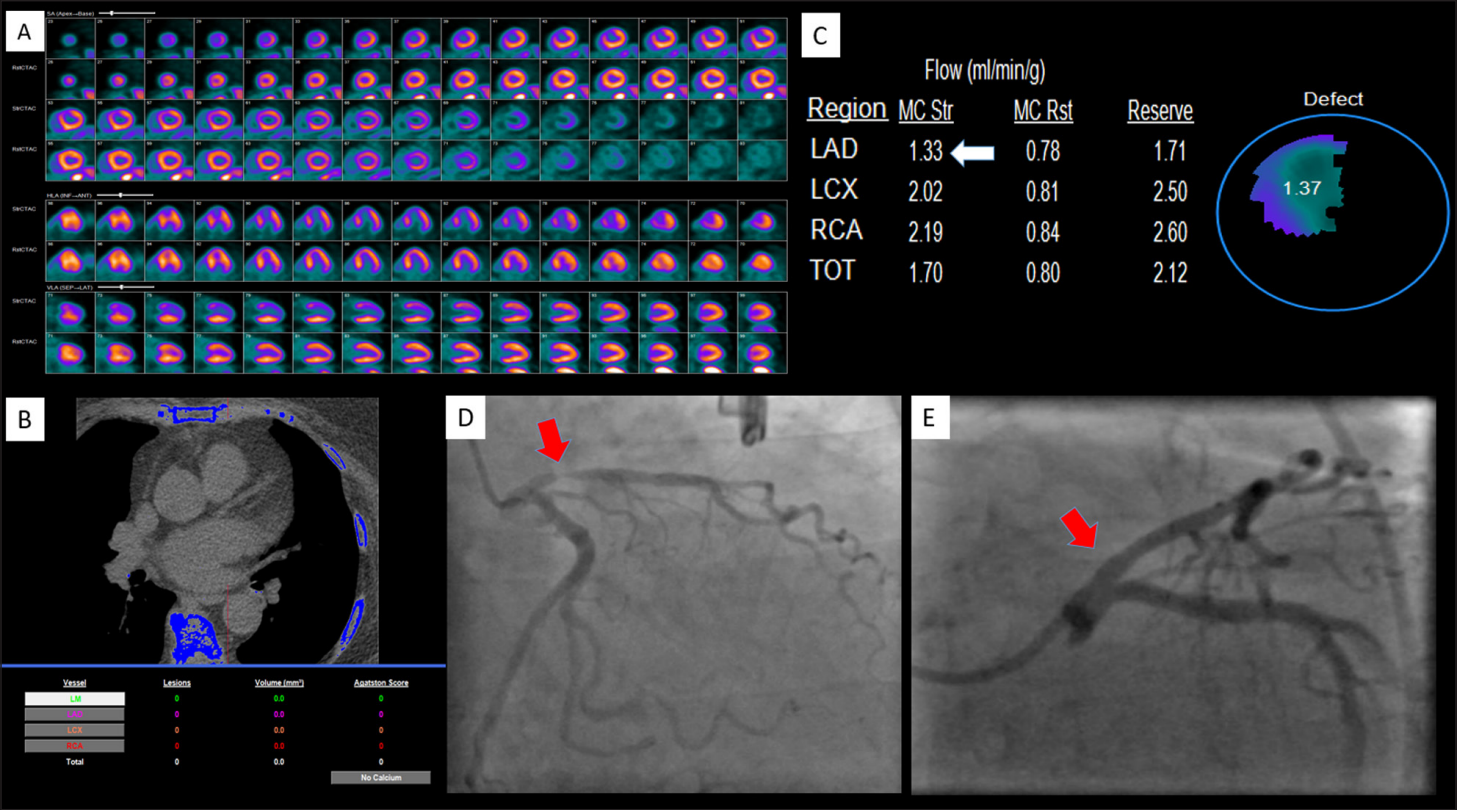
**(A)** PET perfusion images reveal a large, moderate to severe, reversible, myocardial perfusion defect in the LAD coronary artery vascular territory. **(B)** The calcium score was zero. **(C)** Myocardial blood flow analysis revealed reduced peak stress blood flow in the LAD territory but preserved global myocardial flow reserve. **(D)** Invasive angiographic images reveal severe stenosis in the proximal LAD that was **(E)** treated successfully with a drug-eluting stent. PET: positron emission tomography; LAD: left anterior descending artery.

